# Emergency management and nursing care of a patient with moxifloxacin-induced anaphylactic shock complicated with Takotsubo syndrome

**DOI:** 10.1186/s12872-026-06264-z

**Published:** 2026-08-06

**Authors:** Shuqing Jiang, Aihua Jiang, Yongjun Ying, Chunxia Chen, Xian Tian, Xialing Dai

**Affiliations:** 1 Department of General Internal Medicine, Zhejiang Provincial Tongde Hospital, Hangzhou, Zhejiang 320012 China; 2 Department of Nursing, Zhejiang Provincial Tongde Hospital, Hangzhou, Zhejiang 320012 China; 3 Department of Cardiovascular, Zhejiang Provincial Tongde Hospital, Hangzhou, Zhejiang 320012 China

**Keywords:** Anaphylactic shock, Takotsubo syndrome (stress cardiomyopathy), Emergency nursing, Adverse drug reaction (ADR)

## Abstract

**Background:**

Takotsubo syndrome (TTS) is an acute cardiac condition marked by reversible left ventricular dysfunction, with clinical features that resemble acute coronary syndrome but occur without significant coronary artery stenosis. Its development is strongly linked to catecholamine-mediated toxicity. Inflammatory mediators released during allergic responses may further amplify catecholamine secretion; however, drug-induced anaphylactic shock accompanied by TTS has only been rarely documented.

**Case Presentation:**

A 31-year-old female experienced simultaneous anaphylactic shock and TTS (EF 31%) after receiving intravenous moxifloxacin. Emergency management involved adrenaline, corticosteroids, and antihistamines, together with PICCO-guided circulatory support (target MAP ≥ 65 mmHg) and combined antiplatelet/anticoagulant therapy. A family-based “3-min emergency response” training program and a graded rehabilitation protocol were carried out. Before discharge, ADRB1 genetic testing was completed, which identified the GG genotype, and beta-blocker treatment was then started accordingly. The patient’s EF increased to 45% within 48 h, recovered completely within 10 days, and remained stable throughout 6-month pregnancy follow-up under multidisciplinary care, including dynamic EF monitoring ≥ 55% and anticoagulant adjustment.

**Conclusion:**

Moxifloxacin may contribute to TTS through allergic-sympathetic pathways, making careful recognition of overlapping symptoms necessary. Important approaches for lowering recurrence risk include family training, gene-guided therapy, and multidisciplinary cooperation. This study offers useful insight into the care of drug-related TTS, highlighting the need for allergy assessment before drug administration and continued follow-up care. This study was approved by the Medical Ethics Committee of Zhejiang Provincial Tongde Hospital (Ethics Approval No: 2025-Research-069), and the patient provided written informed consent for the use of anonymized case data for academic research.

**Clinical trial number:**

MR-33-25-027765.

## Background

Anaphylactic shock may cause widespread release of inflammatory mediators, and the subsequent administration of epinephrine during emergency resuscitation may also induce substantial catecholamine secretion, both of which are strongly involved in the pathogenesis of Takotsubo syndrome (TTS). Some medications (e.g., β-agonists) may likewise promote the onset of TTS through comparable mechanisms [1, 2]. Anaphylactic shock may increase vascular permeability and disrupt the balance between myocardial oxygen supply and demand, which overlaps with the pathophysiological processes of TTS; in addition, the positive inotropic action of epinephrine used during emergency treatment may further worsen myocardial injury [3, 4].

However, cases in which drug-induced anaphylaxis directly precipitates TTS remain uncommon [2, 4], and moxifloxacin-associated events have been reported only rarely. Because allergic manifestations may obscure the clinical features of TTS and specific biomarkers are lacking, these cases are easily misdiagnosed [4]. This article describes a rare case of a 31-year-old female who developed anaphylactic shock complicated by TTS after intravenous moxifloxacin administration, with the aim of providing a clinical reference for diagnosis, treatment, and nursing care.

### General information

A 31-year-old female (body weight: 51 kg) was admitted to the hospital because of “fever accompanied by cough and expectoration for the past 4 days”. The maximum recorded body temperature was 38.9 °C, and family members reported a recent history of *Mycoplasma pneumoniae* infection. Self-treatment with cephalosporins and acetaminophen had produced no clear improvement. Physical examination on admission showed a blood pressure of 110/93 mmHg, heart rate of 112 beats/min, body temperature of 38.3 °C, SpO_2_ of 97%, and coarse breath sounds over both lungs. Acute bronchitis was considered as the initial diagnosis. The patient had no previous history of allergies or cardiovascular disease.

### Clinical deterioration

On the day of admission, two minutes after intravenous infusion of moxifloxacin (manufacturer: Sichuan Kelun Pharmaceuticals, batch number: N23110607), the patient abruptly developed extremity numbness, skin flushing, dyspnea, a sense of impending death, and hypotension, with blood pressure falling rapidly to 74/44 mmHg, together with vomiting and agitation. Immediate management of anaphylactic shock was performed, including intravenous bolus epinephrine 1 mg, intravenous methylprednisolone 40 mg, intramuscular chlorphenamine 10 mg, and rapid fluid resuscitation. Twenty minutes later, the patient’s impaired consciousness became more severe, and blood pressure varied between 74 and 135 mmHg systolic and 44 and 79 mmHg diastolic. A further dose of epinephrine 1 mg was given intravenously, and the patient was then transferred to the intensive care unit (ICU).

### Diagnostic confirmation

After ICU admission, the patient was somnolent, with cold and clammy skin. The electrocardiogram (ECG) showed sinus tachycardia with widespread T-wave inversion, while serial follow-up revealed no typical progressive changes consistent with ST-segment elevation myocardial infarction. Echocardiography demonstrated impaired left ventricular systolic function (ejection fraction [EF] 31%) with apical ballooning. High-sensitivity troponin I increased to 4.912 µg/L, and chest CT indicated pulmonary edema. Following multidisciplinary consultation, moxifloxacin-induced anaphylactic shock complicated by Takotsubo syndrome (TTS) was confirmed. Coronary angiography was not performed because of hemodynamic instability and typical apical ballooning observed on echocardiography. Based on the InterTAK Diagnostic Score [5], the patient obtained 50 points (female 25 points + physical stress 13 points + no ST-segment depression 12 points), supporting a high likelihood of TTS.

### Treatment

#### Early ICU management

After diagnostic confirmation, goal-directed hemodynamic therapy guided by PICCO (pulse index continuous cardiac output) monitoring was started. Invasive arterial pressure was continuously monitored through a left radial artery catheter, and the following therapeutic targets were established: mean arterial pressure (MAP) 65–70 mmHg, central venous pressure (CVP) 8–12 mmHg, extravascular lung water index (EVLWI) < 10 mL/kg, and stroke volume variation (SVV) < 13% [6]. A staged fluid resuscitation approach was used, beginning with a 500 mL bolus of Ringer’s solution administered at 15 mL·kg^–1^·h^–1^, followed by dynamic adjustment of the infusion rate according to PICCO parameters. Total fluid intake during the first 24 h was restricted to 2000 mL to prevent further aggravation of pulmonary edema [7].

For low cardiac output syndrome, an integrated “volume-cardiac function-oxygen metabolism” management model was applied. On ICU admission, the patient’s cardiac index (CI) was 2.16 L/min/m^2^, and dobutamine was given as a continuous infusion at 1 µg·kg^–1^·min^–1^ to enhance myocardial contractility. Follow-up echocardiography after 48 h demonstrated marked improvement in left ventricular ejection fraction (LVEF), increasing from 31% at ICU admission to 45% (Δ + 14%); systemic vascular resistance index (SVRI) declined from 2929 to 2100 dyn·s/cm^5^; serum lactate decreased from 2.9 to 1.5 mmol/L; and central venous oxygen saturation (ScvO_2_) remained above 70%.

Simultaneous measures were implemented to prevent and treat complications: hypokalemia was corrected, with serum potassium increasing from 2.4 to 4.2 mmol/L, and non-invasive ventilation was used to improve oxygenation. During the treatment period, the patient developed right femoral vein thrombosis, which resolved after 48 h of anticoagulant therapy.

#### Thromboprophylaxis, prevention, and stepwise rehabilitation

An individualized antithrombotic plan was formulated according to the 2023 European Society of Cardiology (ESC) anticoagulation guidelines [8]. Because the patient had concurrent right femoral vein thrombosis, dual antiplatelet loading doses (aspirin 300 mg + clopidogrel 300 mg as a single oral dose) were given during the acute phase, followed by maintenance treatment with aspirin 100 mg once daily and clopidogrel 75 mg once daily, combined with subcutaneous low-molecular-weight heparin 5000 U every 12 h. Ultrasound follow-up confirmed resolution of the right femoral vein thrombus within 48 h. Anti-Xa activity was maintained at 0.6–1.0 U/mL, and the international normalized ratio (INR) was controlled at 1.5–2.0 with daily coagulation assessment; no major bleeding events were observed.

A progressive rehabilitation strategy was carried out. During the acute stage, immobilization combined with passive activity was initiated: after right femoral vein thrombosis was identified on ICU Day 2, the right lower limb was immobilized and elevated, while the left lower limb underwent intermittent pneumatic compression (IPC) therapy (> 18 h/day) together with ankle pump exercises (3 times daily, 5–10 min per session) to support venous return [9]. After thrombus resolution on Day 4, the patient was gradually advanced to sitting in bed, with the head of the bed elevated > 45°, 3 times daily, 15 min per session. On Day 5, under assistance from medical staff, the patient completed bedside sitting and short-distance walking (3 min per session), while vital signs and psychological status were continuously monitored to prevent blood pressure instability and excessive oxygen consumption.

### Continuity of care and follow-up

The patient was transferred from the ICU to the general ward after 6 days and was discharged after a total hospital stay of 10 days. At discharge, repeat echocardiography showed that the left ventricular ejection fraction (LVEF) had improved to > 55%, suggesting recovery of cardiac function to the normal range. Before discharge, ADRB1 Arg389Gly polymorphism testing was conducted, and the GG genotype was identified. The medical team, together with clinical pharmacists, formulated an individualized medication plan: oral metoprolol (25 mg twice daily) was prescribed, and the patient was advised not to discontinue treatment or alter the dose independently to prevent sympathetic nervous system rebound after abrupt withdrawal. If pregnancy was planned, preconception cardiac assessment (echocardiography and Holter monitoring) was recommended, with medication adjustment under combined cardiology and obstetrics follow-up.

A family-integrated “3-min emergency response” protocol was developed. For grade 1 allergic reactions (cutaneous symptoms: flushing, numbness), family members were instructed to stop the suspected causative drug immediately, give oral loratadine 10 mg within 3 min, and continue observation for 30 min. For grade 2 reactions (respiratory or circulatory impairment), family members were trained to administer intramuscular epinephrine 0.3 mg without delay, contact emergency medical services (120), and arrange urgent transfer [10]. Family members received training in epinephrine injection technique (mid-outer thigh, needle depth ≥ 25 mm) and practiced repeatedly to support rapid administration [10]. A portable emergency kit containing epinephrine, loratadine, and an emergency contact card was supplied, with monthly inspection of medication expiry dates and replacement of drugs approaching expiration.

At the 2-month follow-up, no arrhythmia or recurrent cardiac dysfunction was detected. At 6 months after discharge, the patient confirmed pregnancy, and a joint cardiology-obstetrics follow-up program was initiated: echocardiography (assessment of EF and wall motion) and NT-proBNP measurements were arranged every 4 weeks to exclude occult cardiac dysfunction. Considering the hypercoagulable state during pregnancy, enoxaparin (40 mg once daily) was administered subcutaneously until 36 weeks of gestation, with anti-Xa activity monitoring (target: 0.2–0.4 IU/mL) [11]. A psychiatrist evaluated the GAD-7 anxiety scale (threshold < 5, indicating no clinically significant anxiety symptoms) every 2 months [12], together with mindfulness-based stress reduction sessions (3 sessions per week, 20 min per session) to preserve emotional stability. During pregnancy, monthly LVEF assessment (maintained at ≥ 55%) and 24-h Holter monitoring (premature ventricular contractions < 5%/24 h) revealed no TTS recurrence or cardiac dysfunction.

## Discussion

This case report presents a 31-year-old female who developed anaphylactic shock after intravenous moxifloxacin administration and was subsequently diagnosed with Takotsubo syndrome (TTS), presenting as typical apical ballooning. The main characteristics of this case are two aspects: (1) moxifloxacin as the precipitating drug has been rarely documented in the available literature; and (2) the patient later conceived successfully and delivered at full term after the initial TTS episode, without adverse pregnancy outcomes related to the TTS history. The following discussion addresses four key areas: pathophysiological mechanisms, comparison with previous literature, iatrogenic reflections, and clinical implications.

### Mechanisms of TTS in this case: anaphylactic shock triggering catecholamine storm and myocardial stunning

This case demonstrates a clear pathophysiological sequence. Moxifloxacin induced an IgE-mediated type I hypersensitivity reaction, resulting in mast cell degranulation and the release of large amounts of inflammatory mediators, which subsequently increased vascular permeability and reduced effective circulating blood volume. Anaphylactic shock itself activated the stress response, causing massive endogenous catecholamine release; at the same time, exogenous epinephrine given during resuscitation further increased circulating catecholamine levels. Together, endogenous and exogenous catecholamines produced a sudden and marked rise in circulating catecholamine concentrations.

Excessive catecholamine concentrations cause myocardial stunning through several mechanisms: (1) direct myocardial toxicity, characterized by intracellular calcium overload and contractile protein degradation; (2) microvascular dysfunction, leading to insufficient myocardial perfusion because of coronary microvascular spasm; and (3) endothelial injury, produced by the combined damaging effects of inflammatory mediators and catecholamines on the vascular endothelium [3, 13, 14]. These mechanisms may explain why the patient developed a markedly reduced left ventricular ejection fraction of 31% and showed typical apical ballooning morphology despite the absence of significant coronary artery stenosis.

Importantly, beyond the endogenous catecholamine surge caused by anaphylactic shock, exogenous epinephrine may also directly promote myocardial stunning through β_2_ receptor-mediated negative inotropic effects. In recent years, the ATAK (Adrenaline, Takotsubo, Anaphylaxis, Kounis syndrome) complex has been introduced to describe the clinical continuum involving epinephrine, TTS, and Kounis syndrome during anaphylactic shock [15]. The present case fits well within this pathophysiological framework and adds clinical evidence from a drug-allergy context to support the ATAK complex.

### Literature comparison: uniqueness and clinical value of the present case

The mechanisms described above suggest that both anaphylactic shock and epinephrine exposure may precipitate TTS. In recent years, this clinical association has been supported by several case reports. In 2025, Le et al. described a 58-year-old female who developed anaphylactic shock after oral amoxicillin-clavulanate administration and later presented with TTS (ejection fraction 35%) 22 h after receiving intramuscular epinephrine and intravenous maintenance treatment outside the hospital [16]. Khan et al. reported a 71-year-old female who developed anaphylactic shock due to pistachio allergy and manifested TTS (ejection fraction 45%) within minutes after intramuscular epinephrine administration [17].

### Compared with the above cases, the distinctiveness of the present case is reflected in three main aspects

First, moxifloxacin, a fourth-generation fluoroquinolone, is extensively used in clinical practice; however, allergic reactions, once they occur, may be highly severe [18]. Moxifloxacin-induced anaphylactic shock complicated by TTS remains uncommon in the published literature, and this case offers an important safety reminder for clinicians when prescribing this class of drugs.

Second, the timing of TTS onset in this case coincided with the shock phase. TTS developed during resuscitation for anaphylactic shock and was confirmed by echocardiography immediately after ICU admission, which differs from the delayed onset reported by Le et al. [16] and the rapid onset described by Khan et al. [17]. This temporal pattern indicates that TTS in the present case may have been caused by the combined effects of anaphylactic shock and epinephrine administration, rather than by either factor alone.

Third, this case presents a relatively complete nursing management experience. The above-mentioned reports were mainly clinical case descriptions and lacked a systematically summarized nursing perspective. In contrast, the present case details PICCO-guided hemodynamic management, graded rehabilitation, family-based emergency training, and multidisciplinary pregnancy follow-up, which forms the main value that distinguishes it from purely clinical reports.

### Iatrogenic reflection: unexpected complications arising from routine treatment

This case clearly illustrates an “iatrogenic cascade”: routine anti-infective treatment with moxifloxacin triggered anaphylactic shock, which required epinephrine administration and subsequently induced TTS, followed by complications such as low cardiac output, pulmonary edema, and thrombosis, ultimately transforming simple bronchitis into a complex clinical crisis. This sequence suggests that even routinely used medications may still lead to serious clinical consequences.

Because moxifloxacin is commonly used in clinical practice for community-acquired pneumonia, its routine and standardized use may easily reduce clinical vigilance. The present case suggests that allergy risk assessment before drug administration should not be treated as a formality, and close observation should be maintained throughout infusion [19]. For patients with allergy history or high-risk features, skin testing or alternative agents may be considered before drug use. As the first-line rescue drug for anaphylactic shock, epinephrine may produce different cardiovascular effects depending on the administered dose and route.

In the present case, the initial 1 mg intravenous bolus of epinephrine was given according to resuscitation guidelines for severe anaphylactic shock. However, because exogenous epinephrine is recognized as a potential trigger for TTS, the dosing strategy requires further refinement. The optimization of epinephrine administration, such as through fractionated intramuscular injection or individualized dosing while maintaining resuscitation efficacy, deserves further investigation.

Furthermore, this iatrogenic cascade suggests that during resuscitation for anaphylactic shock, medical teams may focus heavily on immediate hemodynamic instability and unintentionally miss potential myocardial injury. When blood pressure fluctuations and impaired consciousness occur, early indicators of TTS, including electrocardiographic (ECG) abnormalities and increased troponin levels, may be obscured. The present case indicates that routine ECG monitoring and cardiac biomarker testing should be performed during and after anaphylactic shock resuscitation, so that possible concomitant TTS can be identified early and delayed diagnosis caused by overlapping symptoms can be avoided.

### Pregnancy management: multidisciplinary collaboration and continuity of care

Another important value of this case is that the patient, a 31-year-old woman of childbearing age, confirmed pregnancy 6 months after the initial TTS episode and successfully completed pregnancy and delivery under multidisciplinary collaborative management, without TTS recurrence or cardiac dysfunction.

TTS mainly affects postmenopausal women, and its occurrence in women of childbearing age is relatively rare [20]. Although peripartum TTS is uncommon, it has been described in the literature, and physical and psychological stress during pregnancy and delivery, together with rapid changes in estrogen levels, may act as possible triggers for TTS [21]. For women of childbearing age with a previous history of TTS, unified recommendations for subsequent pregnancy management are currently unavailable, and published evidence remains limited. The management experience from this case suggests that pregnancy care in this patient population should focus on three key stages: preconception, antenatal, and peripartum management.

The central aim of preconception management is to assess cardiac function and optimize medication strategies. Although present evidence supporting the use of ADRB1 genotype to guide beta-blocker therapy remains limited [22], the central mechanism of TTS involves catecholamine toxicity caused by sympathetic overactivation; therefore, cardiac assessment before pregnancy and medication adjustment under multidisciplinary follow-up have important clinical significance.

The focus of antenatal management is cardiac function surveillance and thromboprophylaxis. The increases in blood volume and cardiac output during pregnancy markedly increase cardiac workload, making regular cardiac function assessment necessary in patients with a history of TTS [21]. At the same time, the hypercoagulable state associated with pregnancy substantially raises the risk of thromboembolism [23]. For patients with a previous history of TTS complicated by thrombotic events, indications for anticoagulation should be actively assessed, and low-molecular-weight heparin has shown a favorable safety profile during pregnancy [24]. Therefore, systematic cardiac monitoring and individualized anticoagulation management during pregnancy have considerable clinical relevance in this patient population.

In addition, psychological support and emotional regulation should not be neglected. TTS is often precipitated by emotional or physical stress, and hormonal changes during pregnancy, together with the marked physiological alterations during delivery, may act as potential triggering factors [24]. Strengthening emotional counseling and stress management alongside routine obstetric care may help lower the potential risk of TTS recurrence.

In summary, for women of childbearing age with a previous history of TTS, pregnancy is not an absolute contraindication; however, a full-cycle continuity-of-care model covering preconception assessment, antenatal monitoring, delivery management, and postpartum follow-up should be established. Multidisciplinary collaboration remains essential for ensuring maternal and fetal safety.

## Conclusions

This case report presents a rare case of moxifloxacin-induced anaphylactic shock complicated by TTS, with pathogenesis involving the synergistic effects of allergic reactions and catecholamine storm, consistent with the pathophysiological framework of the ATAK complex. This case is notable for the offending drug (moxifloxacin), the temporal pattern of onset (concurrent with the shock phase), and pregnancy management after recovery. The present case indicates that even routine antibiotic therapy may initiate a serious iatrogenic cascade; individualized treatment, including gene testing-assisted medication exploration, family-integrated emergency training, and multidisciplinary continuity of care, may represent effective strategies for reducing the risk of TTS recurrence. For women of childbearing age with a history of TTS, a successful pregnancy, under close multidisciplinary collaborative management, is feasible.

## Data Availability

The datasets used and/or analysed during the current study are available from the corresponding author on reasonable request.
